# Orf Virus 002 Protein Targets Ovine Protein S100A4 and Inhibits NF-κB Signaling

**DOI:** 10.3389/fmicb.2016.01389

**Published:** 2016-09-13

**Authors:** Daxiang Chen, Zewei Zheng, Bin Xiao, Wei Li, Mingjian Long, Huiqin Chen, Ming Li, Daniel L. Rock, Wenbo Hao, Shuhong Luo

**Affiliations:** ^1^Institute of Antibody Engineering, School of Biotechnology, Southern Medical UniversityGuangzhou, China; ^2^State Key Laboratory of Organ Failure, Guangdong Provincial Key Laboratory of Tropical Disease Research, School of Biotechnology, Southern Medical UniversityGuangzhou, China; ^3^Guangdong Provincial Key Laboratory of Tropical Disease Research, School of Public Health, Southern Medical UniversityGuangzhou, China; ^4^Department of Pathobiology, College of Veterinary Medicine, University of Illinois at Urbana–Champaign, UrbanaIL, USA

**Keywords:** ORFV002, NF-κB, yeast two-hybrid, S100A4, inhibition, interaction

## Abstract

Orf virus (ORFV), a member of *Parapoxvirus*, has evolved various strategies to modulate the immune responses of host cells. The ORFV-encoded protein ORFV002, a regulator factor, has been found to inhibit the acetylation of NF-κB-p65 by blocking phosphorylation of NF-κB-p65 at Ser^276^ and also to disrupt the binding of NF-κB-p65 and p300. To explore the mechanism by which ORFV002 regulates NF-κB signaling, the understanding of ORFV002 potential binding partners in host cells is critical. In this study, ovine S100 calcium binding protein A4 (S100A4), prolyl endopeptidase-like (PREPL) and NADH dehydrogenase (ubiquinone) 1 alpha subcomplex 8 (NDUFA8) were found to interact with ORFV002 based on the yeast two-hybrid (Y2H) assay using a cDNA library derived from primary ovine fetal turbinate cells (OFTu). GST pull-down and bidirectional co-immunoprecipitation assay results demonstrate that ORFV002 interacts with S100A4 directly. Following the pEGFP-ORFV002 (p002GFP) transfection, we found that cytoplasmic S100A4 translocates into the nucleus and co-localizes with ORFV002. Furthermore, the inhibitory effect of ORFV002 on NF-κB signaling was significantly restored by S100A4 knock-down phenotype, suggesting that ovine S100A4 participates in the ORFV002-mediated NF-κB signaling. These data demonstrate that ORFV002 inhibits the NF-κB activation through its interaction with S100A4 along with its nucleus translocation.

## Introduction

Orf virus (ORFV), a type of *parapoxvirus* belonging to the poxvirus family, causes orf disease which mainly affects sheep, goats, and other ruminants (Hosamani et al., 2009). The primary invasion sites of ORFV are keratinocytes and epithelial cells in the oral mucosa where some typical symptoms, such as contagious ecthyma, contagious pustular dermatitis, and scabby mouth, occur (Haig, 2006). Persistent infection with ORFV is often due to the virus evading immunological surveillance. ORFV encodes immunomodulating proteins to avoid or adapt to host immune responses by various strategies. For example, the ORFV132 gene encodes a viral VEGF protein to stimulate the proliferation of vascular endothelial cells and promote vascular permeability, the ORFV125 gene encodes a Bcl-2-like inhibitor of apoptosis to facilitate viral replication, and the ORFV117 gene encodes an IL-10-like protein that exerts immunosuppressive activity on the host immune response (Savory et al., 2000; Imlach et al., 2002; Westphal et al., 2007).

Of these, ORFV002, 024 and 121 function as modulating molecules in the NF-κB pathway (Diel et al., 2010, 2011a,b). ORFV002 is a protein of 117 amino acids encoded by a gene near the left terminal of the ORFV genome. It is an early late gene that transcribed at low level at early time and markedly increased from 6 to 12 h post-infection. After translation, ORFV002 accumulates in the nucleus (Diel et al., 2011a). Our previous studies have revealed that ORFV002 could inhibit NF-κB signaling through the interaction with NF-κB-p65 and decreasing TNF-α and wild-type-virus-induced acetylation of NF-κB-p65 by blocking Ser^276^ residue phosphorylation. Further study showed that the N-terminal 52 amino acids of ORFV002 play a role in inhibiting both the acetylation and phosphorylation of NF-κB-p65 (Diel et al., 2011a; Ning et al., 2013). However, the binding partner (s) of ORFV002 which mediates the downstream effects on NF-κB signaling is not clear.

The yeast two-hybrid assay is one of the most powerful tools in evaluating direct protein–protein interactions. A recent study, using the yeast two-hybrid assay, revealed that the viral protein F12 associates with IEV through an interaction with the protein A36 (Johnston and Ward, 2009). We therefore applied the yeast two-hybrid system to explore the direct interacting proteins with ORFV002. The preliminary interaction of ORFV002 with S100A4 was identified.

S100A4, a member of the S100 family, could modulate invasion, metastasis, apoptosis, and differentiation of various malignant tumors through different mechanisms (Ji et al., 2014; Dahlmann et al., 2016). The association of S100A4 with NF-κB was demonstrated in previous investigations. The activation of the NF-κB signaling pathway, phosphorylation of p65 and degradation of IkB by LPS stimulation, increased the expression of S100A4 in human periodontal ligament (Mah et al., 2015). Another study showed that S100A4 increased the proinflammatory and prometastatic action via activation of the transcription factor NF-κB (Haase-Kohn et al., 2011). After secreted, S100A4 activates NF-κB by inducing phosphorylation of IKKα/β, leading to an increased IκBα phosphorylation (Grotterod et al., 2010). The intracellular S100A4 drives migration and invasion of HepG2 cells via the NF-κB-dependent MMP9 signal (Zhang et al., 2013). S100A4 can also promote squamous cell laryngeal cancer Hep-2 cell invasion via NF-κB/MMP-9 signal (Zhang et al., 2014). Thus, S100A4 and NF-κB components could activate each other reciprocally depending on different cell types. In present study, we further knocked down endogenous S100A4 using small interfering RNA (siRNA) to investigate its role in the NF-κB signaling. Our results demonstrate that ORFV002 physically binds to and functions through S100A4 on NF-κB signaling.

## Materials and Methods

### Cells

Ovine fetal turbinate (OFTu) cells were prepared as described previously (Ning et al., 2013), and cultured in minimal essential medium (MEM) supplemented with 10% fetal bovine serum (Hyclone, USA), 100 μg/ml streptomycin, 100 U/ml penicillin, 50 μg/ml gentamicin and 2 mM _L_-glutamine. Human embryonic kidney cell line 293T (HEK293T) was cultured in Dulbecco’s modified Eagle’s medium (DMEM) supplemented with 10% fetal bovine serum.

### Plasmid Construction

The plasmid pEGFP-ORFV002 (p002EGFP) was constructed as described previously (Ning et al., 2013). The synthetic ORFV002 full-length DNA was cloned into the *EcoRI* and *BamHI* sites of the bait vector pGBKT7 to generate plasmids pGBKT7-ORFV002. Ovine S100A4 was amplified from the cDNA library of OFTu cells and sub-cloned into the pCMV-Tag2B and pGEX4T-3 vectors to construct the eukaryotic and prokaryotic expression plasmids. The primer sequences were synthesized as follows:

pGBKT7-ORFV002-Fw : CgaattcATGACTCCTACTTCTCG AGAAT (EcoRI)
pGBKT7-ORFV002-Rv: CggatccATTAGTAGTGGTAGTCT AGC (BamHI)
pCMV-Tag2B-S100A4-Fw: TATAggatccATGGCATACCCC CTGGAGAAG (BamHI)
pCMV-Tag2B-S100A4-Rv: ATCActcgagTCACTTTTTCCG GGGTTGCT (XhoI)
pCMV-Tag2B-S100A4-Fwkozak: TATAggatccGCCACCATG GCGTACCCCCTGG (BamHI)
pGEX4T-3-S100A4-Fw: TATAggatccATGGCATACCCCCTG GAGAAG (BamHI)
pGEX4T-3-S100A4-Rv: ATCActcgagGATCACTTTTTCCG GGGTTGCT (XhoI)
Restriction sites were in lowercase.

### Yeast Two-Hybrid System

The yeast two-hybrid assay process is shown in **Supplementary Figure S1**. SMART (Switching Mechanism At 5′ end of the RNA Transcript) cDNA synthesis technology was used to map cDNA libraries derived from OFTu cells. The cDNA pools, together with linearized prey vector pGADT7-Rec were transformed into yeast strain Y187 to generate the libraries, utilizing the highly potent homologous recombination machinery of *Saccharomyces cerevisiae*. The BD-bait plasmid pGBKT7-ORFV002 was generated and transformed into the yeast strain Y2HGold. The toxicity and auto activation of the bait was tested on SD/-Trp liquid medium and SDO (SD/-Trp), SDO/X (SD/-Trp/X-a-Gal), and SDO/X/A (SD/-Trp/X-a-Gal/AbA) plates. The library strain and ORFV002-bait-expressing reporter strain (Y2HGold) were mixed and left overnight and selected on DDO/X/A (SD/-Leu/-Trp/X-a-Gal/AbA) plates. Positive colonies were confirmed on QDO/X/A (SD/–Ade/–His/–Leu/–Trp/X-a-Gal/AbA) plates. Finally, the plasmids was extracted from the blue colonies, sequenced and analyzed through NCBI BLAST searches.

### GST Pull-Down

GST pull-down assay aims to detect the direct binding of eukaryotic form of ORFV002 to prokaryotic GST-tagged S100A4. The plasmid pGEX4T-3 or pGEX4T-3-S100A4 was transformed into *Escherichia coli* strain BL21/DE3 to induce expression of GST or GST-fusion protein by adding 1.0 mM IPTG for 16 h at 18°C overnight. The collective bacteria pellet was disrupted by lysozyme, followed by repetitive freeze-thawing and sonication. The soluble proteins were obtained by centrifugation at 10,000 × *g* for 20 min at 4°C. The Beaver beads TM GSH kit (Beaver Nano Technology, China) was used to purify the GST-fused or GST proteins from the supernatant according to the manufacturer’s instructions. Plasmids pEGFP-N1 and p002EGFP were transfected into HEK293T cells, respectively, using lipofectamine 2000 reagent (Invitrogen, USA) following the manufacturer’s instructions, and then incubated for 24 h. RIPA lysis buffer was added to the cells after washing and plating on ice for 15 min. The cell lysate was centrifuged at 14000 × *g* for 15 min and the supernatant containing GFP or 002GFP proteins was collected. 5 ng purified proteins were incubated with 25 μl Glutathione Sepharose 4B beads for 3 h at 4°C. The beads were washed three times and incubated with supernatant (500 ng total protein) containing GFP or 002GFP proteins overnight at 4°C. The beads were washed three times again, and the bead-bound protein complexes were detected by western blot using anti-GFP or anti-GST antibodies (ABclonal, USA).

### Co-immunoprecipitation

Co-immunoprecipitation was performed to determine the direct or indirect interaction of ORFV002 and S100A4 in eukaryotic forms. HEK293T cells were co-transfected with either p002EGFP, pEGFP-N1 and pCMV-Tag2B-S100A4, or either pCMV-Tag2B-S100A4, pCMV-Tag2B and p002EGFP. The cells were washed twice with cool PBS and lysed with 1 ml cool lysis buffer with 1 mM phenylmethylsulfonyl fluoride (PMSF) for 10 min on ice at 24 h post-transfection, followed by centrifugation at 14000 × *g* for 10 min. Three micrograms of antibodies against GFP-tag (Santa Cruz Biotechnology; USA) or FLAG-tag (ABclonal; USA), or isotype IgG incubated with 30 μl pre-treated Beaver Beads Protein A/G (Beaver Nano Technology, China) for 2 h at 4°C. The obtained supernatant was immunoprecipitated with the washed Beads-antibody overnight at 4°C. After washing and centrifugation, the pellet was examined by western blot using anti-GFP or anti-FLAG antibodies.

### Immunofluorescent Analysis

Ovine fetal turbinate cells were cultured on glass-covered slips and co-transfected with 1.5 μg pCMV-Tag2B-S100A4 and either 1.5 μg pEGFP-N1 or p002EGFP. Cells were washed and permeabilized with 0.025% TritonX-100/PBS after fixing with 4% paraformaldehyde for 15 min at 24 h post-transfection. After blocking with 1% bovine serum albumin (BSA)/PBS, the cells were incubated with anti-FLAG primary antibody (1:300 in PBST containing 1% BSA) for 2 h at room temperature, washed three times and incubated with red fluorescence secondary antibody anti-mouse Alexa Fluor 594 (1:1000 in PBST containing 1% BSA) for 1 h at room temperature. After washing and staining with DAPI (0.1 μg/ml, 10 min), the cells were examined by laser scanning confocal microscopy (LSM700; Zeiss, Germany).

### siRNA Transfection

The short siRNA was constructed by Shanghai Genepharma, with sequences specifically targeted to ovine S100A4 gene (**Table 1**). The plasmids and siRNAs were transfected into OFTu cells using Lipofectamine 2000 (Life Technology; USA) according to the manufacturer’s protocol. The effects of siRNA interferon were examined by western blot.

**Table 1:** The small interfering RNA (siRNA) sequences.

siRNA	Sense	Antisense
S100A4-i153	5′-UCCACAAGUACUCGGGCAATT-3′	5′ -UUGCCCGAGUACUUGUGGATT-3′
S100A4-i348	5′-GCAUCGCCAUGAUGUGCAATT-3′	5′ -UUGCACAUCAUGGCGAUGCTT-3′
Negative control	5′-UUCUCCGAACGUGUCACGUTT-3′	5′ -ACGUGACACGUUCGGAGAATT-3′

### Dual-Luciferase Assay

The OFTu cells were cultured in 24-well plates (7 × 10^4^ cells/well) and co-transfected with 250 ng pNF-κBLuc, 50 ng pRLTK, either 300 ng pEGFP-N1 or p002EGFP and 40 pmol siRNA. Twenty-four hours post-transfection, cells were treated with 20 ng/ml of TNF-α for 6 h. The cells then were lysed and NF-κB activity was measured with the Dual-Luciferase Reporter Assay System (Promega, USA).

### Western Blots

Western blots were used to assess the effect of siRNA, GST-pull down, co-IP, and the extraction of cytoplasmic and nuclear protein. A ProteoJet cytoplasmic and nuclear protein extraction kit (Fermentas; USA) was used to extract the cytoplasmic and nuclear protein fractions according to the manufacturer instructions. The samples were resolved by SDS-PAGE in 12% gels, followed by blotting to PVDF membranes. Then the membranes were blocked with 5% non-fat milk and probed with respective primary antibodies. After washing with TBST (0.1% Tween-20 in TBS), the blots were incubated with secondary goat anti-rabbit (mouse) IgG-(HRP). Finally, they were washed three times with TBST and developed using a chemiluminescent substrate.

### Statistics

All procedures were performed at least three times. Data are presented as mean ± standard deviation and subjected to the student’s *t*-test and One-Way ANOVA.

## Results

### Yeast Two-Hybrid Screening Results

**FIGURE 1:**
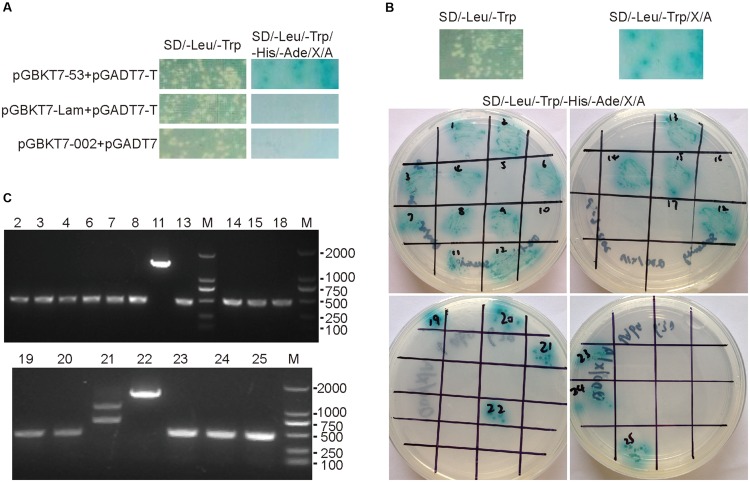
**Yeast-two hybrid screening results of ORFV002.** The mated cultures were spotted on synthetic dropout SD/-Trp/-Leu/-His/-Ade/X-α-Gal/AbA agar plates. Positive interactions were confirmed by growth and blue staining. The SD/-Leu/-Trp control plates were used to confirm the presence of both plasmids in yeast cultures (left of 2A and 2B upper portion). **(A)** Mated diploids containing pGBKT7-53 and pGADT7-T were used as positive control. Either pGBKT7-Lam and pGADT7-T or pGBKT7-ORFV002 and pGADT7 were used as negative control. **(B)** Mating the Y187 library of OFTu with Y2HGold harboring pGBKT7-ORFV002 and screening for interaction on synthetic dropout SD/-Trp/-Leu/X-α-Gal/AbA agar plates. The positive clones were confirmed on SD/-Trp/-Leu/-His/-Ade/X-α-Gal/AbA agar plates. **(C)** The results of PCR amplification for the inserted fragments in the positive diploid yeast clones are shown. Except for the clones No.11, 21, and 22, the length of the inserted fragments in the clones were the same, about 500 bp.

In order to identify the interacting partners for the ORFV002 protein, we conducted a yeast two-hybrid screen using cDNA libraries derived from OFTu cells. The toxicity and auto transcriptional activation of the fusion construct pGBKT7-ORFV002 were excluded according to the manufacturer’s manual. The library was screened using the plasmid pGBKT7-ORFV002 as a “bait.” Twenty-one positive clones containing three genes with different functions were obtained and sequenced from the screen (**Figure 1**; **Table 2**). The sequence similarity analysis showed that they were the ovis aries S100A4, prolyl endopeptidase-like (PREPL) and NADH dehydrogenase (ubiquinone) 1 alpha subcomplex 8 (NDUFA8). S100A4, whose function is related to the NF-κB signaling pathway, was present in 18 clones. According to previous studies, S100A4 can stimulate metastatic progression through both intracellular and extracellular functions, while extracellular S100A4 can activate NF-κB in some cell lines (Boye et al., 2008; Grotterod et al., 2010; Siddique et al., 2013). Thus, ovine S100A4 was selected to further characterization.

**Table 2:** BLAST results of 21 positive clones by yeast two-hybrid screening of ovine fetal turbinate (OFTu) cDNA library with ORFV002 as bait.

Genes	Clones	Function	Similarities in Blast	GenBank accession number
Ovis aries prolyl endopeptidase-like, transcript variant 3 (PREPL), mRNA	2	Serine-type peptidase activity	98%	XM_004005984.1
Ovis aries S100 calcium binding protein A4 (S100A4), mRNA	18	Positive regulation of I-κB kinase/NF-κB cascade	99%	XM_004002526.1
Ovis aries NADH dehydrogenase (ubiquinone) 1 alpha subcomplex 8, 19 kDa (NDUFA8), mRNA	1	Transfer of electrons from NADH to the respiratory chain	99%	XM_004005667.1

### Extracellular and Intracellular Interaction of ORFV002 with S100A4

**FIGURE 2:**
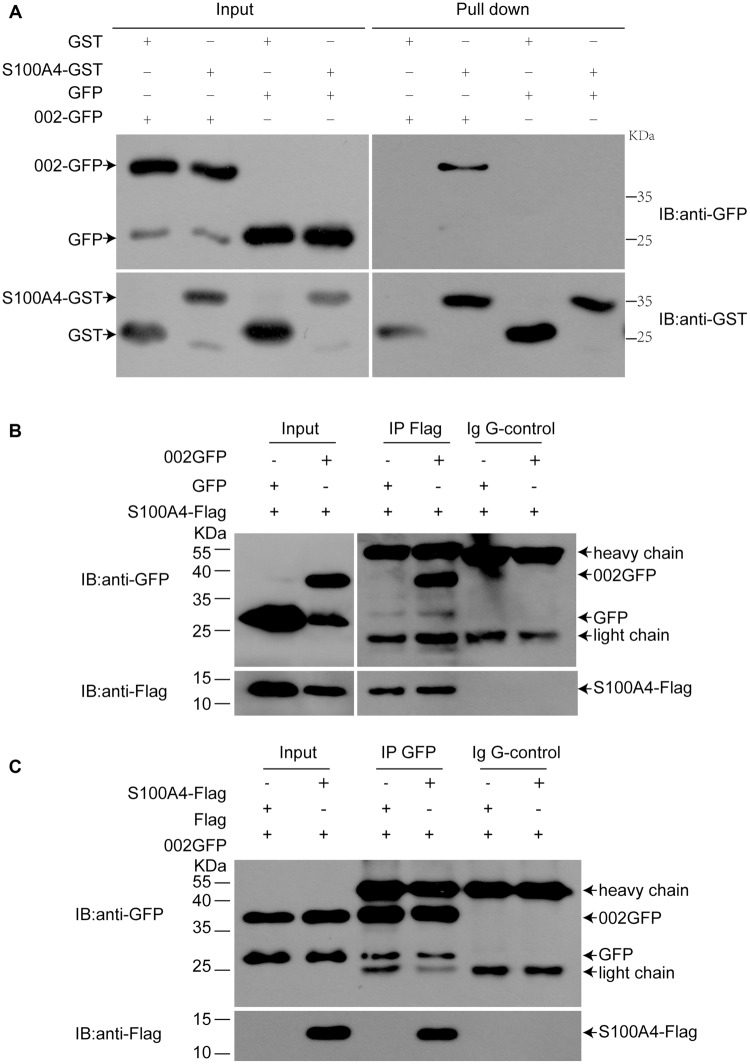
**Confirmation of the interaction between ovine S100A4 and ORFV002 using GST pull-down and co-immunoprecipitation. (A)** GST pull-down assays were performed with p002GFP or pEGFP-N1 transfected HEK293T cell lysates as prey and GST-S100A4 as bait. Immunoblotting was performed using antibodies detecting GFP or GST. 002GFP could be pulled-down by GST-S100A4 but not by GST, while GFP was not pulled-down by either GST-S100A4 or GST. **(B)** HEK293T cells were co-transfected with either pEGFP-N1 or p002EGFP and pCMV-Tag2B-S100A4 and subjected to immunoprecipitation with anti-FLAG antibody or isotype IgG. In immunoprecipitates using anti-FLAG antibody, 002GFP was detected. **(C)** GFP immunoprecipitation of co-transfected HEK293T cells co-immunoprecipitated S100A4. Light chain = light chain of IgGs, heavy chain = heavy chain of IgGs.

To confirm the interaction of ORFV002 with S100A4 outside or in the cells, GST pull-down and co-immunoprecipitation assays were performed. GFP or GFP-fusion proteins were acquired from HEK293T cells transfected with pEGFP-N1 or pEGFP-002, while GST or GST-S100A4 was acquired via prokaryotic protein expression and purification. Either 002GFP or GFP was incubated with GST-S100A4 or GST. We showed that 002GFP could be pulled down by GST-S100A4 but not by GST, while GFP was not pulled down by either GST-S100A4 or GST (**Figure 2A**). This result indicated that ORFV002 physically interacts with ovine S100A4. In addition, bidirectional co-immunoprecipitation assays were performed after co-transfection with p002EGFP and pCMV-Tag2B-S100A4 in HEK293T cells. A reproducible 002GFP and S100A4-FLAG interaction was identified (**Figures 2B,C**). 002GFP was detected in samples immunoprecipitated using FLAG-tag specific antibody (**Figure 2B**), but not with an isotype Ig G antibody (negative control). Similarly, S100A4-FLAG was detected in 002GFP immunoprecipitated samples, but not GFP immunoprecipitated samples (**Figure 2C**). The co-immunoprecipitation results demonstrate that ORFV002 could bind to ovine S100A4 in natural form.

### ORFV002 Drives the Translocation of Ovine S100A4 to the Nucleus and Co-localizes with S100A4

**FIGURE 3:**
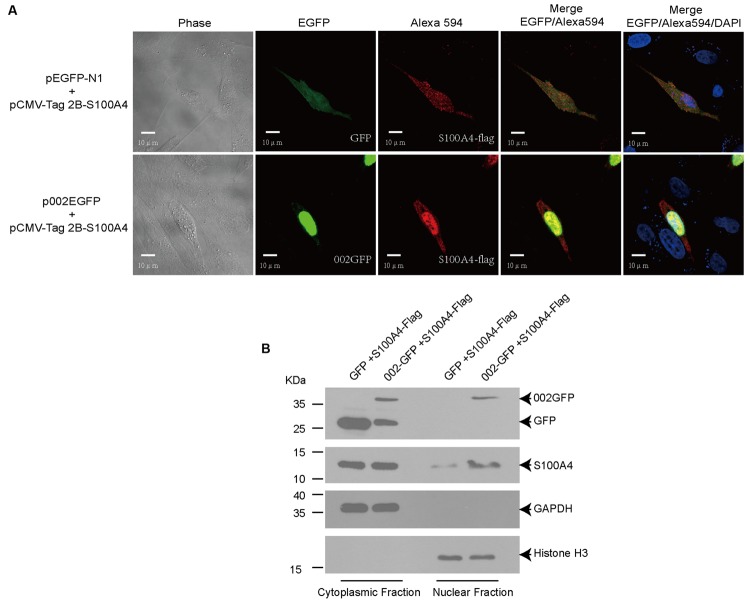
**Co-localization of ORFV002-GFP and S100A4-FLAG. (A)** OFTu cells were transiently co-transfected with plasmids pCMV-Tag2B-S100A4 and p002EGFP (or pEGFP-N1, as control). Red fluorescence indicates the localization of S100A4 using the anti-FLAG primary antibody and Alexa Fluor 594 secondary antibody. The 002GFP fusion protein was localized by green fluorescence. The blue fluorescence shown in the cell nucleus indicates proteins stained by DAPI. **(B)** Cytoplasmic and nuclear protein fractions were extracted from **(A)** and detected by western blot. The membranes were probed with antibodies against GFP (top panel), FLAG-tag (second panel), GAPDH (third panel), or histone H3 (bottom panel).

To further test the interaction between ORFV002 and ovine S100A4, an immunofluorescence analysis was performed after co-transfection with either pEGFP-N1 or p002EGFP and pCMV-Tag2B-S100A4 in OFTu cells. After 24 h, the cells were probed with an antibody against FLAG-tag, and subsequently examined by confocal microscopy (**Figure 3A**). As described in a previous study (Diel et al., 2011a), ORFV002 localized mainly in the nucleus. Ovine S100A4 exhibited a homogeneous and diffuse distribution in the cytoplasm when co-expressed with GFP. Co-expression of S100A4 and ORFV002 resulted in an altered distribution pattern of ovine S100A4, which was intensively localized in the nucleus with small quantity of distribution in the cytoplasm. Moreover, the cytoplasmic and nuclear protein fractions were extracted to detect the co-localization of ORFV002 and S100A4 in the nucleus. As shown in **Figure 3B**, compared to GFP-expressing cells, the expression of ORFV002-GFP markedly increased nuclear accumulation of S100A4. Together, the results indicate that cytoplasmic S100A4 translocated into the nucleus and co-localized with 002GFP.

### Knock Down of S100A4 Using siRNA Affects ORFV002 Function

**FIGURE 4:**
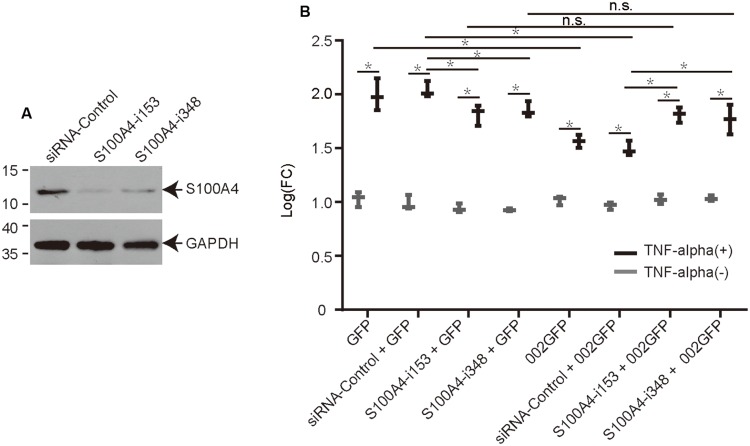
**The effect of ovine S100A4 knockdown on the modulation of NF-κB by ORFV002. (A)** The effect of two siRNAs targeting different sites of the S100A4 gene is shown by western blot. **(B)** OFTu cells were co-transfected with plasmids pRLTK, pNF-κBLuc, different siRNAs or no siRNA and either pEGFP-N1 or p002EGFP. Firefly and sea pansy luciferase activities were measured at 6 h after treatment with TNF-α and expressed as relative fold changes in luciferase activity. (*n* = 3, *p < 0.05, n.s, no significance).

Interaction of ORFV002 with S100A4 was inferred as an underlying mechanism for the inhibitory effect of ORFV002 on the NF-κB signaling pathway. Two siRNAs targeting different sites of the ovine S100A4 gene were designed to knock down S100A4 in OFTu cells (**Figure 4A**). The luciferase activities in cells stimulated by TNF-α were significantly higher than in cells without TNF-α treatment in every two paired groups, indicating that NF-κB signaling was activated in response to TNF-α. Compared to the levels for the siRNA control sample, the knock-down of S100A4 resulted in a significant increase in luciferase activity in samples with 002GFP at 6 h after treatment with TNF-α (*p* < 0.001, **Figure 4B**). But there was no difference between group S100A4-i153 + 002GFP and group S100A4-i153 + GFP or group S100A4-i348 + 002GFP and group S100A4-i348 + GFP. The results indicate that ovine S100A4 is involved in the action of ORFV002 on the NF-κB signaling. Meanwhile, the activity of NF-κB was reduced in S100A4-i153 + GFP and S100A4-i348 + GFP sample, compared to the group siRNA-control + GFP, which was in line with the function of S100A4 in modulating NF-κB pathway (Boye et al., 2008; Grotterod et al., 2010; Yang et al., 2013). Comparing the panel of GFP and 002GFP, we concluded that ORFV002 inhibits the activity of NF-κB, which was consistent with our previous report (Diel et al., 2011a).

## Discussion

In this study, the yeast two-hybrid system was performed to screen protein (s) interacting with ORFV002. After three rounds of screening, 21 positive clones were identified, containing three proteins which are likely to interact with ORFV002 in OFTu cells. Of these interactive proteins, ovis aries S100 calcium binding protein A4 was deemed the reliable for its frequency, emerging in 18 of the clones. We performed GST pull-down assay and co-immunoprecipitation in HEK293T cells to confirm the interaction between ORFV002 and ovine S100A4. Further, the cytoplasmic S100A4 translocated into the nucleus and co-localized with ORFV002 in OFTu cells, which supports this conclusion.

The S100A4 protein, a member of the S100 family, has been associated with cancer metastasis. This function has been demonstrated in extracellular studies and animal experiments in rodents (Garrett et al., 2006; Boye and Maelandsmo, 2010). In a previous study, extracellular S100A4 specifically activates NF-κB in several human cancer cell lines via the classical NF-κB pathway (Boye et al., 2008). Another study revealed that S100A4 can activate NF-κB by means of phosphorylation of IKKα/β and the subsequent increase of IκBα phosphorylation (Grotterod et al., 2010). The activation was shown to be independent of the putative S100 protein receptor RAGE and the Ser/Thr kinases MEKK1, NIK and AKT in II-11b cells. The intracellular S100A4 also drives migration and invasion of HepG2 cells via NF-κB-dependent MMP9 signal (Zhang et al., 2013). These results indicate the importance of S100A4 and the divergence of its function in different cells (Siddique et al., 2013).

ORFV002 can inhibit NF-κB by blocking the acetylation of p65. We speculate that ORFV002 binds to ovine S100A4 and functions to inhibit NF-κB signaling, although there may be additional pathways involved. Therefore, we used a dual-luciferase assay to detect the activity of NF-κB with and without siRNA targeting ovine S100A4. After knock down of endogenous S100A4, the activity of NF-κB with GFP was reduced by RNA interference, which was consistent with a prior study (Yang et al., 2013). One might deduce that endogenous S100A4 can activate NF-κB signaling and that the interaction of the two proteins inhibits S100A4’s role in the NF-κB signal pathway. However, the 002GFP-mediated inhibition of NF-κB was restored when S100A4 was knocked down by siRNA and not in GFP alone with siRNA (**Figure 4B**). The result showed that ovine S100A4 is a partner for ORFV002 to exert an inhibitory effect on the NF-κB signaling. In the present study, we failed to identify the physical interaction between NF-κB-p65 and ORFV002 using a yeast two-hybrid assay, although their interaction had previously been confirmed by co-immunoprecipitation (Diel et al., 2011a), suggesting that the interaction between ORFV002 and NF-κB-p65 may be indirect. In addition, S100A4 is translocated into the nucleus from the cytoplasm along with the expression of ORFV002 (**Figure 3B**). Miranda et al. (2010) reported that S100A4 could translocate into the nucleus with the treatment of IL-1β in human articular chondrocytes, a process that required the post-translational modification of sumoylation. Nuclear S100A4, as a transcriptional regulator, was bound to the promoter region of MMP-13 and regulate the expression of the MMP-13. As indicated in previous studies, three ORFV encoded proteins, ORFV002, ORFV024, and ORFV121, are all inhibitors of NF-κB signaling, but function at different points in the host inflammatory response (Diel et al., 2010, 2011a,b). While ORFV002 inhibits acetylation of p65, ORFV024 can inhibit activation of IKKs, and ORFV121 binds to and inhibits the phosphorylation of NF-κB-p65. Whether S100A4 affects the function of ORFV024 and ORFV121 on NF-κB-p65 signaling, this hypothesis needs further investigation.

In our previous work, we found that the N-terminal segment of ORFV002 is responsible for the inhibition of NF-κB-p65 acetylation (Ning et al., 2013). Thus, the next phase of our research will seek to characterize the interaction of the N-terminal part of ORFV002 with S100A4. A single protein often has multiple biological functions through binding with different partners during different physiological or developmental stages of the cells, forming functional protein heterodimers or protein complexes. Among the ORFV002 interactomics, S100A4 is one of the most competitive binding targets, as indicated by the number of positive clones in our yeast two-hybrid screening. However, we cannot neglect the other two potential ORFV002 interacting proteins, PREPL and NDUFA8. Further investigations are required to demonstrate whether they are genuine ORFV002 binding partners.

## Conclusion

The present study further characterizes the molecular mechanism by which ORFV002 inhibits the NF-κB activation and may provide possible new insight into the role of NF-κB inhibitors encoded by ORFV in mediating S100A4-centered NF-κB program.

## Author Contributions

SL, WH, ML, and DR participated in design of the study. DC, ZZ, WL, BX, ML, and HC performed the experiments. DC, BX, WL, and SL analyzed the data and wrote the manuscript. All authors read and approved the final manuscript.

## Conflict of Interest Statement

The authors declare that the research was conducted in the absence of any commercial or financial relationships that could be construed as a potential conflict of interest.

## Supplementary Material

FIGURE S1 **A flow diagram of yeast two-hybrid assay system**. Total RNA was extracted from OFTu cells and used to construct cDNA libraries using SMART cDNA synthesis technology. The cDNA pools, together with linearized prey vector pGADT7-Rec, were transformed into yeast strain Y187 to generate the libraries. The bait plasmid pGBKT7-ORFV002 were constructed, transformed into the yeast strain Y2HGold and were tested for toxicity and autoactivation. When cultures of the two transformed strains are mixed together overnight, they mate to create diploids. Diploid cells were selected on DDO/X/A plate and positive colonies were confirmed on QDO/X/A plates. Finally, the plasmids were extracted from the blue colonies and sequenced.
