# Corrigendum: Orf Virus 002 Protein Targets Ovine Protein S100A4 and Inhibits NF-κB Signaling

**DOI:** 10.3389/fmicb.2017.00160

**Published:** 2017-02-07

**Authors:** Daxiang Chen, Zewei Zheng, Bin Xiao, Wei Li, Mingjian Long, Huiqin Chen, Ming Li, Daniel L. Rock, Wenbo Hao, Shuhong Luo

**Affiliations:** ^1^Institute of Antibody Engineering, School of Biotechnology, Southern Medical UniversityGuangzhou, China; ^2^State Key Laboratory of Organ Failure, Guangdong Provincial Key Laboratory of Tropical Disease Research, School of Biotechnology, Southern Medical UniversityGuangzhou, China; ^3^Guangdong Provincial Key Laboratory of Tropical Disease Research, School of Public Health, Southern Medical UniversityGuangzhou, China; ^4^Department of Pathobiology, College of Veterinary Medicine, University of Illinois at Urbana–ChampaignUrbana, IL, USA

**Keywords:** ORFV002, NF-κB, yeast two-hybrid, S100A4, inhibition, interaction

There is an error in the Funding statement. The correct number for National High Technology Research and Development Program 863 is No. 2014AA020904. The authors apologize for this error and state that this does not change the scientific conclusions of the article in any way.

## Conflict of interest statement

The authors declare that the research was conducted in the absence of any commercial or financial relationships that could be construed as a potential conflict of interest.

