# Supplementary figures and images for: Orf Virus 002 Protein Targets Ovine Protein S100A4 and Inhibits NF-κB Signaling
Source: Front Microbiol. 2016 Sep 13;7:1389. doi: 10.3389/fmicb.2016.01389 (PMC5020088; doi:10.3389/fmicb.2016.01389)

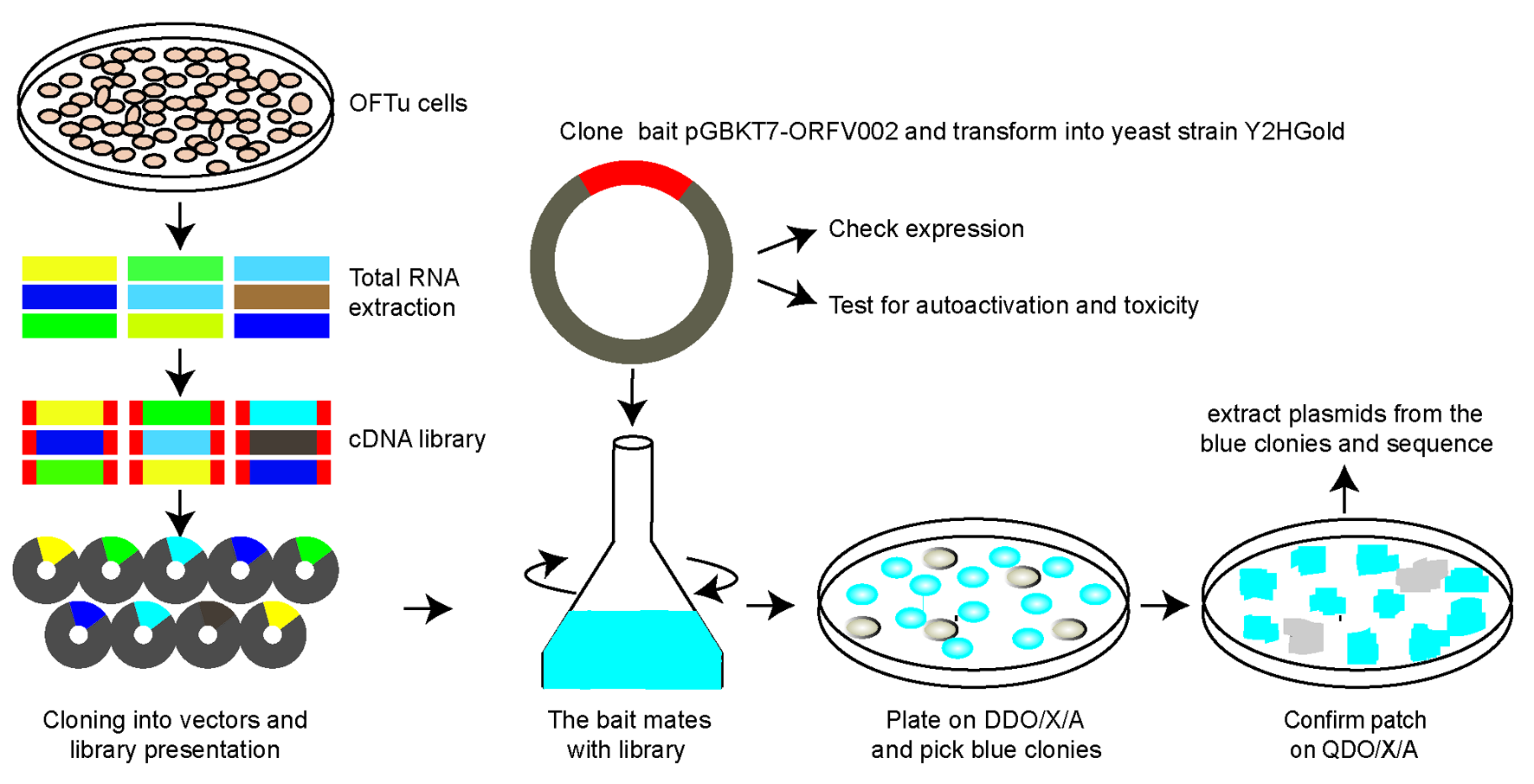

Supplement: FIGURE S1 — A flow diagram of yeast two-hybrid assay system. Total RNA was extracted from OFTu cells and used to construct cDNA libraries using SMART cDNA synthesis technology. The cDNA pools, together with linearized prey vector pGADT7-Rec, were transformed into yeast strain Y187 to generate the libraries. The bait plasmid pGBKT7-ORFV002 were constructed, transformed into the yeast strain Y2HGold and were tested for toxicity and autoactivation. When cultures of the two transformed strains are mixed together overnight, they mate to create diploids. Diploid cells were selected on DDO/X/A plate and positive colonies were confirmed on QDO/X/A plates. Finally, the plasmids were extracted from the blue colonies and sequenced. [file Image_1.TIF]
